# Quantifying the accuracy of deformable image registration for cone‐beam computed tomography with a physical phantom

**DOI:** 10.1002/acm2.12717

**Published:** 2019-09-21

**Authors:** Richard Y. Wu, Amy Y. Liu, Tyler D. Williamson, Jinzhong Yang, Paul G. Wisdom, Xiaorong R. Zhu, Steven J. Frank, Clifton D. Fuller, Gary B. Gunn, Song Gao

**Affiliations:** ^1^ Department of Radiation Physics The University of Texas MD Anderson Cancer Center Houston TX USA; ^2^ Department of Radiation Oncology The University of Texas MD Anderson Cancer Center Houston TX USA

**Keywords:** adaptive plan, CBCT image registration, deformable image registration, deformable phantom

## Abstract

**Purpose:**

Kilo‐voltage cone‐beam computed tomography (CBCT) is widely used for patient alignment, contour propagation, and adaptive treatment planning in radiation therapy. In this study, we evaluated the accuracy of deformable image registration (DIR) for CBCT under various imaging protocols with different noise and patient dose levels.

**Methods:**

A physical phantom previously developed to facilitate end‐to‐end testing of the DIR accuracy was used with Varian Velocity v4.0 software to evaluate the performance of image registration from CT to CT, CBCT to CT, and CBCT to CBCT. The phantom is acrylic and includes several inserts that simulate different tissue shapes and properties. Deformations and anatomic changes were simulated by changing the rotations of both the phantom and the inserts. CT images (from a head and neck protocol) and CBCT images (from pelvis, head and “Image Gently” protocols) were obtained with different image noise and dose levels. Large inserts were filled with Mobil DTE oil to simulate soft tissue, and small inserts were filled with bone materials. All inserts were contoured before the DIR process to provide a ground truth contour size and shape for comparison. After the DIR process, all deformed contours were compared with the originals using Dice similarity coefficient (DSC) and mean distance to agreement (MDA). Both large and small volume of interests (VOIs) for DIR volume selection were tested by simulating a DIR process that included whole patient image volume and clinical target volumes (CTV) only (for CTVs propagation).

**Results:**

For cross‐modality DIR registration (CT to CBCT), the DSC were >0.8 and the MDA were <3 mm for CBCT pelvis, and CBCT head protocols. For CBCT to CBCT and CT to CT, the DIR accuracy was improved relative to the cross‐modality tests. For smaller VOIs, the DSC were >0.8 and MDA <2 mm for all modalities.

**Conclusions:**

The accuracy of DIR depends on the quality of the CBCT image at different dose and noise levels.

## 1 INTRODUCTION

Image guidance is widely used in radiation therapy. Many modern linear accelerators are equipped with on‐board imaging that can acquire kilo‐voltage (kV) cone beam computed tomography (CBCT).^1^ CBCT is widely used for patient alignment and more recently for adaptive treatment planning. Targets and organs are known to change position and shape during fractionated radiotherapy.^2^ Deformable image registration (DIR) software has gained acceptance for managing contour propagation, dose tracking, and related issues over the course of such therapy. Deformable image registration enables users to automatically adjust the treatment planning contours drawn on the initial planning CT scan to account for anatomic changes observed on subsequent CT or CBCT images and to modify the plans as needed. For routine clinical use, the contour propagation process must have acceptable accuracy.

Commercial DIR software programs including MIM Maestro (MIM Software Inc., Cleveland, OH, USA), Velocity (Varian Medical Systems, Palo Alto, CA, USA) and RayStation (RaySearch Laboratories, Stockholm, Sweden) are being used for propagating contours with CBCT images in clinical practice.^3^, ^4^, ^5^ The performance of DIR depends on numerous variables such as type of algorithm, implementation of that algorithm, and image modality and quality.^6^ The clinical stability of DIR is also influenced by factors such as the method of regularization^6^ and user experience.^7^ Several methods have been used to evaluate DIR algorithms, the three most common are contour outline comparison, landmark tracking, and simulating deformation with a phantom.^8^ Validating and commissioning DIR are complex because of the lack of systematically documented processes for doing so. Currently, means of validating the accuracy of deformable registration are being investigated at academic institutions. Until the technology advances to allow production of a standard testable deformable phantom, the most common way to review deformation at present is by visual verification,^9^ including tissue/voxel intensity overlay, viewing the deformable warp map, and displaying the difference map between two registered images. The American Association of Physics in Medicine (AAPM) recommends that formal image registration quality assurance (QA) programs be implemented at individual facilities. The program should include commissioning image registration and fusion software to ensure the accuracy of the tools used.^6^ Understanding the optimization approach used by the user's DIR system is essential to appreciate how it converges, its limitations, and its potential pitfalls. Last year, the AAPM task group 132 reported^6^ a new, downloadable virtual phantom to test DIR accuracy and recommended using either a digital phantom or a physical phantom for DIR tests. However, the digital phantom does not facilitate end‐to‐end testing of DIR systems, in particular, facilitating the selection of optimal imaging parameters for DIR systems. In addition, reports have shown that the validation procedure is more complex for digital phantoms.^10^ No standardized physical phantom is available to date that can be used to test DIR accuracy under same or different imaging modalities [e.g., CT vs. CT, CT vs CBCT, CBCT vs CBCT, magnetic resonance imaging (MRI) vs MRI, MRI vs. CT and positron emission tomography (PET) vs PET or MRI] and different image quality conditions.

The quality of images obtained with cone‐beam geometry is known to be inferior to that of regular CT images because of the large solid angle receiving scattered radiation.^11^ Increased scatter from the patient obstructs the signal, degrading CBCT image quality compared with standard CT, resulting in blurred images and changes in CT numbers.^12^ For kV CBCT, up to 2.5 times more photons arriving at a detector behind a normal‐sized patient body are scattered as compared to fan beam CT.^12^, ^13^ The accuracy of DIR systems for CBCT images under various levels of noise and dose has not been well studied. Decreases in soft tissue CT number intensity have been noted from increased beam hardening and truncation of images due to smaller field of view (FOV). Some physical phantoms have been developed to assess the accuracy of DIR.^4^, ^10^, ^14^, ^15^, ^16^, ^17^, ^18^, ^19^, ^20^ Despite the existence of many methods to independently validate DIR systems, none have been standardized and all demand a great deal of time and resources.

We previously presented our work on the development of a physical phantom (Wuphantom, US patent application) that can be used to seamlessly quantify the accuracy of a DIR system.^21^ Here we used this physical phantom to evaluate the accuracy of DIR for CBCT with various scanning protocols and levels of image quality. Our goal in this testing was to evaluate the accuracy of DIR in (a) cross‐modality registration (CT‐vs‐CBCT), (b) same‐modality registration (CBCT‐vs‐CBCT and CT vs CT), and (c) these CBCT registrations with different‐sized volume of interest (VOIs).

## 2 METHODS

### 2.A Phantom

**Figure 1:**
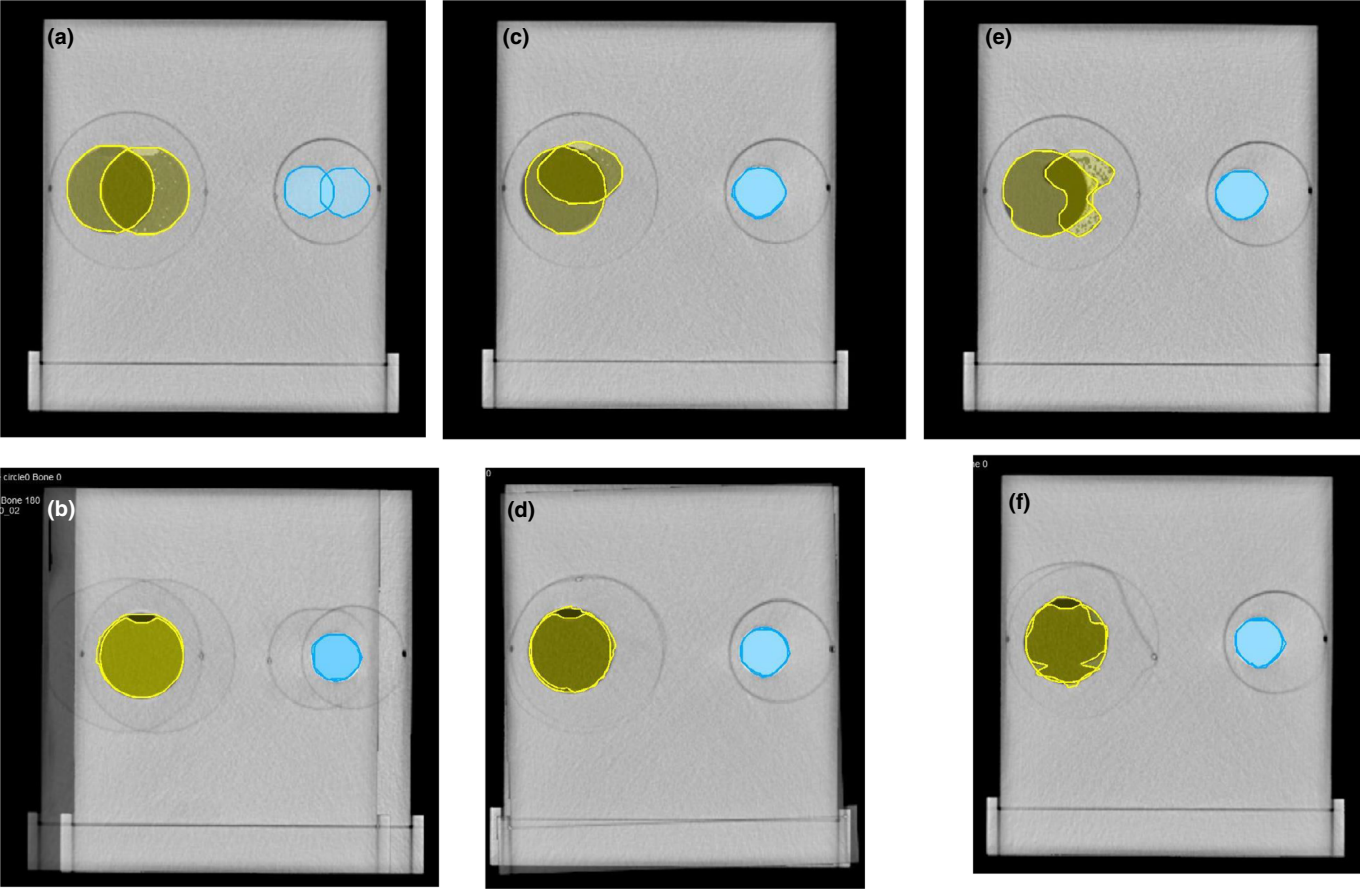
Illustration of contour deformation and location changes. (a) Overlay of contours prior to DIR. Circle (Yellow) rotation = 180°, bone (Light‐blue) rotation = 180°. (b) Overlay of deformed contour circle and bone from (a). This is contour location change of 20mm. (c) Overlay of contours prior to DIR. Oval (Yellow) rotation = 90°, bone rotation = 0°. (d) Overlay of the deformed Oval shape contour and bone from (c). (e) Overlay of contours prior to DIR. Irregular (Yellow) rotation = 180°, bone rotation = 0°. (f) Overlay of deformed Irregular shape contour and bone from (e). DIR, deformable image registration.

We previously designed a physical phantom that can be used to test the accuracy of DIR. The phantom is acrylic and includes a variety of inserts to simulate different tissue shapes and properties. Deformations and anatomic changes can be simulated by changing the rotations of both the phantom and the inserts. Three large cavity inserts were created in different shapes: circle, oval, and irregular, simulating deformed contours from the original circle (Fig. 1). The oval shape represents commonly deformed contours, and the irregular shapes simulate irregularly deformed contours. For DIR testing, the inserts are rotated to simulate contour changes in both shape and location compared with the reference circle. Each of these large insert cavities was filled with Mobil DTE oil (density 0.95 g/mL) to represent soft tissue. A smaller cavity on the right side of the phantom was filled with bone plug (CB2 30%) from RMI (Gammex, Inc.), which has a density of 1.33 g/mL to simulate bone and changes in bone location.

### 2.B Phantom image acquisition

**Figure 2:**
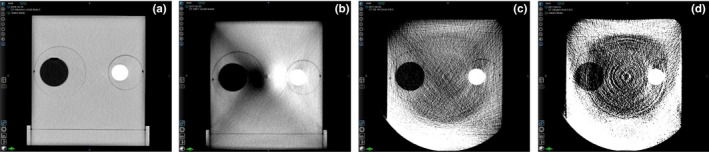
Comparison of images obtained with various techniques under the same viewing conditions (abdomen window/level) in Velocity. (a) Image of original CT scan obtained with the head and neck imaging protocol. (b) Image of CBCT pelvis imaging protocol with beam hardening artifacts. (c) Image of CBCT head imaging protocol with truncation artifacts and increased noise level. (d) Image of CBCT gently imaging protocol with further enhanced noise and artifacts level. CBCT, cone‐beam computed tomography.

CT images of the Wuphantom were acquired with a Siemens Definition Edge CT scanner [Fig. 2(a)]. All CBCT scans were obtained on a Truebeam linear accelerator with an OBI System (Varian Medical Systems, Inc., CA, USA) [Figs. 2(b) and 2(d)]. The CT image has a voxel resolution of 0.98 × 0.98 × 2 mm. Scanning was done with an established head and neck CT protocol (35 cm FOV, 120 kVp, 2.0 mm slice thickness, and 300 mA). The CBCT images were acquired with pelvis, head and "Image Gently” protocols to represent images with various noise and dose levels, with image quality levels ranging from best to worst in a typical clinical environment. Image Gently protocol gives much less radiation exposure to patients at the cost of increased image noise level, mostly used for patient alignment or pediatric patients. Image acquisition variables are shown in Table 1. All images were then transferred to a Velocity Workstation 4.0 (Varian, Inc.).

**Table 1:** Cone‐beam CT and CT image acquisition protocols and variables.

Protocol Name	Tube potential, kVp	mAs	Fan type	Gantry rotation, degrees	Field of view, cm^2^	Measured contrast‐to‐noise‐ratio	Patient dose level (CTDIw), cGy
CBCT Image Gently	80	100	Full	200	25 × 25	0.68	0.09
CBCT Head	100	150	Full	200	25 × 25	1.28	0.32
CBCT Pelvis	125	1050	Half	360	45 × 45	1.91	1.62
CT Head & Neck	120	300	Full	360	50 × 50	2.82	4.09

CBCT, cone‐beam computed tomography.

### 2.C Deformable image registration test

#### 2.C.1 Image registration and contour propagation

For original CT images, contours were delineated for the large and small inserts by using a predefined range of threshold CT numbers before image registration, to provide baseline contours of various shapes for quantitatively validating the DIR. For CBCT images, due to increase noise level and CT number variations, the contours were copied from the original CT images. All of the alignment marks (insert rotation) were set at 0° during acquisition of the reference image set. The secondary images for the DIR accuracy tests were acquired by replacing and rotating the circular and oval‐ and irregular‐shaped inserts, to simulate tissue deformation from a circular shape to another circular shape or a different shape (oval or irregular). Rotating the inserts to different degrees simulates location changes for the contours of interest. Eleven combined contour deformation scenarios that simulate both contour deformation and location changes are shown in Table 2. The small circle (filled with bone plug) had both rotated and non‐rotated conditions, simulating clinical situations in which only soft tissue, and not bone, had deformation. We measured the volume of all inserts from the original CT images.

**Table 2:** Eleven contour deformation scenarios that simulate both contour deformation and location changes.

Phantom, Insert, and Rotation, degrees	Measured Volume of Inserts (cm^3^)	
	Circle/Oval/Irregular	Bone
Circle 0 Bone 0 (Reference image)	100.1	48.1
Circle 90 Bone 45	100.5	48.1
Circle 180 Bone 180	105.4	46.2
Circle 270 Bone 225	98.1	47.3
Oval 0 Bone 0	68.3	46.8
Oval 90 Bone 0	68.9	47.4
Oval 180 Bone 0	68.9	46.9
Oval 270 Bone 0	68.1	47.4
Irregular 0 Bone 0	58.3	48.3
Irregular 90 Bone 0	58.8	48.1
Irregular 180 Bone 0	58.5	47.2
Irregular 270 Bone 0	58.7	46.8

Abbreviations: Circle, large insert of circular shape; Bone, small insert with bone plug; Oval, large insert with oval shape; Irregular, large insert with irregular shape.

We used the rigid registration first and followed by deformable multi‐pass tool in the Velocity software program for the DIR process for all of the selected secondary image sets. Then, all CBCT images were deformed to the corresponding reference CBCT or reference CT images. One setting used a larger VOI (20 cm × 20 cm × 14 cm) for DIR of the entire phantom, to simulate the registration being focused on the entire image volume of the patient, including body structures, organ at risk (OARs), and clinical target volume (CTV). The other setting used a smaller VOI (8 cm × 8 cm × 7 cm) on soft tissue inserts only, simulating deformation focusing on CTV propagations. All of the corresponding contours were propagated onto the secondary image sets.

#### 2.C.2 Image quality

**Figure 3:**
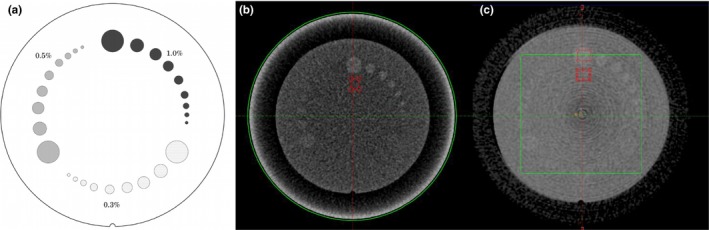
Quantitative measurement of contrast‐to‐noise ratio. (a) Catphan model 604 low‐contrast section. (b) Measurement of 15‐mm diameter circle with 1% contrast object using original CT head & neck protocol. (c) Measurement using CBCT pelvis imaging protocol on same locations. CBCT, cone‐beam computed tomography.

CT and CBCT image quality can be quantified in terms of the contrast‐to‐noise ratio (CNR).^22^ A Catphan with a module containing low‐contrast cylinders (CTP 604) was used to determine the CNR [Fig. 3(a)]. A 15‐mm diameter, 1.0% nominal low‐contrast object was measured for a region of interest (ROI) of 7 mm × 7 mm. The measurement was also obtained in the background area near the object. The measurements were performed for images acquired by the CT head & neck protocol [Fig. 3(b)] and CBCT pelvis protocol [Fig. 3(c)]. The measurement process was also repeated for images obtained with CBCT head and CBCT Image Gently protocols (not displayed). The CNR is defined as:

$$\text{CNR}=\left(\text{ROI}\left(1\%\right)-\text{ROI}\left(\text{bk}\right)\right)/\text{SD}\left(\text{bk}\right).$$

where ROI (1%) represents the mean Hounsfield units (HU) in the ROI of a 15‐mm diameter, low‐contrast object; ROI (bk) represents the mean HU of the adjacent background; and SD (bk) represents the standard deviation of the background.

#### 2.C.3 Accuracy of DIR

Contours can be quantitatively compared by using several metrics. Two commonly used approaches are the Dice similarity coefficient (DSC)^23^ and mean distance to agreement (MDA),^6^, ^24^ also referred to as the mean distance to conformity. The DSC is defined as the ratio of twice the overlap of two structures over the sum of their volumes. This method is widely used in DIR comparisons. The DSC ranges from 0 to 1 according to the degree of match between the two structures; a value of 0 indicates no overlap, and a value of 1 indicates perfect agreement. The MDA is a geometric variable that measures the mean voxel shortest distance from the surface of one structure to another; the ideal is 0 mm.

## 3 RESULTS

**Figure 4:**
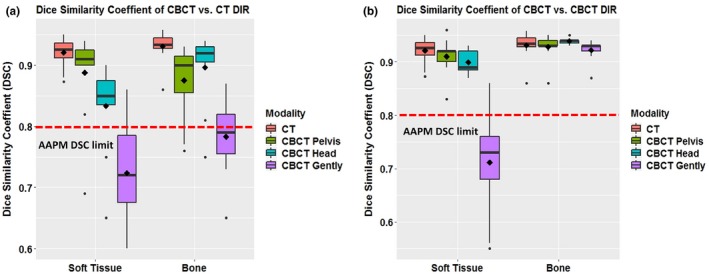
Accuracy of deformable image registration measured with the Dice similarity coefficient (DSC). Box‐and‐whisker plot shows the accuracy of deformable image registration measured with the DSC. The thick horizontal lines represent the median. The diamond indicates the mean value. The lower and upper boundary correspond to the first and third quartiles (the 25th and 75th percentiles). The whiskers show the maximum and minimum values, up to 1.5 times the inter‐quartile range. The dots indicate outliers. (a) Cross‐modality image registration (CBCT vs CT, also showed CT vs CT as a reference). (b) Same‐modality image registration (CBCT vs CBCT, also showed CT vs CT as a reference). CBCT, cone‐beam computed tomography.

**Figure 5:**
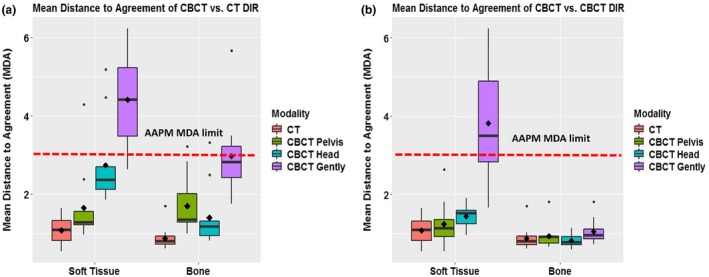
Accuracy of deformable image registration by mean distance to agreement. (a) Cross‐modality image registration from CBCT vs CT (also showed CT vs CT as a reference). (b) Same‐modality image registration from CBCT vs. CBCT (also showed CT vs CT as a reference). CBCT, cone‐beam computed tomography.

The measured CNR values for each imaging protocol were 2.8 (original CT head & neck), 1.91 (CBCT pelvis), 1.28 (CBCT head), and 0.68 (CBCT Image Gently), which correspond to patient dose levels (CTDIw) of 4.09 cGy, 1.62 cGy, 0.32 cGy, and 0.09 cGy (Table 1). Cross‐modality image registration of soft tissue and bone resulted in DSC values (means ± Standard deviation (SD)] of 0.89 ± 0.07 (soft tissue) and 0.88 ± 0.06 (bone) (for CBCT pelvis vs CT); 0.83 ± 0.07 and 0.90 ± 0.06 (for CBCT head vs CT), and 0.72 ± 0.08 and 0.78 ± 0.06 (for CBCT Image Gently vs. CT) [Fig. 4(a)]. For same‐modality image registration, the DSC values were 0.92 ± 0.02 (soft tissue) and 0.93 ± 0.02 (bone) (for CT); 0.91 ± 0.03 and 0.93 ± 0.02 (for CBCT pelvis); 0.90 ± 0.02 and 0.94 ± 0.01 (for CBCT head); and 0.71 ± 0.09 and 0.92 ± 0.02 (for CBCT Image Gently) [Fig. 4(b)]. As for the MDA, cross‐modality registration values (means ± *SD*) were 1.65 mm ± 0.91 (soft tissue) and 1.70 mm ± 0.73 (bone) (for CBCT pelvis vs CT); 2.74 mm ± 1.03 and 1.40 mm ± 0.75 (for CBCT head vs CT), and 4.4 mm ± 1.1 and 2.98 mm ± 0.98 (for CBCT Image Gently vs CT) [Fig. 5(a)]. Same‐modality registration led to MDA values of 1.07 mm ± 0.34 (soft tissue) and 0.88 mm ± 0.29 (bone) (for CT vs CT); 1.24 mm ± 0.55 and 0.93 mm ± 0.30 (for CBCT pelvis); 1.44 mm ± 0.15 and 0.82 mm ± 0.27 (for CBCT head); and 3.8 mm ± 1.48 and 1.05 mm ± 0.3 (for CBCT Image Gently) [Fig. 5(b)].

**Figure 6:**
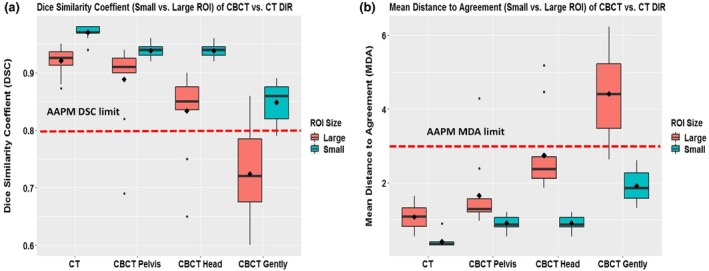
Accuracy of deformable image registration with large and small regions of interest (ROIs) for CT vs CBCT with different imaging protocols. (a) Dice similarity coefficient (DSC) values were higher than the American Association of Medical Physicists (AAPM) limit (red dashed line) for both large and small ROIs in all protocols except CBCT Gently vs CT using large ROIs. (b) Mean distance to agreement (MDA) values were lower than the AAPM limit (red dashed line) for all small ROIs in all protocols.

Comparisons of the accuracy of cross‐modality DIR (DSC and MDA) using large and small VOIs for soft tissue inserts are shown in Fig. 6. The mean ± *SD* DSC and MDA values for small VOIs were 0.97 ± 0.1 (DSC) and 0.4 mm ± 0.17 (MDA) (for CT vs CT); 0.94 ± 0.01 and 0.91 mm ± 0.20 (for CBCT pelvis vs CT); 0.94 ± 0.01 and 0.91 mm ± 0.2 (for CBCT head vs CT); and 0.85 ± 0.04 and 1.92 mm ± 0.44 (for CBCT Image Gently vs CT). The DIR for small VOIs was significantly better than that for larger VOIs in all imaging protocols.

## 4 DISCUSSION

We quantitatively validated the accuracy of a DIR system for CBCT vs CT and CBCT vs. CBCT at different noise and dose levels. AAPM TG 132 specifies that the accuracy of DIR should be reflected in a DSC of 0.8–0.9 and a mean MDA of 2–3 mm. In our test of cross‐modality DIR accuracy of CBCT vs. CT, all results except for those obtained with the CBCT “Image Gently” protocol were within the AAPM recommended levels for both soft tissue and bone, because the objects in CBCT images with “Image Gently” protocol have the lowest contrast compared to CBCT images with other protocols. In our test of same‐modality DIR accuracy of CBCT for various protocols, all results, except for those obtained with the CBCT Image Gently protocol for soft tissue, were acceptable. The same‐modality DIR accuracy of CBCT with “Image Gently” protocol for bone was acceptable, because the image contrast of the bone insert is higher than the contrast of soft tissue insert, and the image contrasts of bone are similar in both reference and the secondary images. One would expect that this DIR would result in reduced registration accuracy when the deformation is large or when the boundaries between structures are not clear; to address this point, we tested the DIR accuracy using both large and small VOIs. Using smaller VOIs provided increased accuracy for all CBCT imaging protocols. These findings imply that CBCT can be used for adaptive planning, dose tracking, and so on, but only with selected imaging techniques that provide adequate CNR.

Singhrao et al., in their study of DIR algorithms for evaluating MV and kV imaging of a head and neck phantom, found that the presence of artifacts on CBCT images is problematic for algorithms that focus strongly on image similarity.^16^ We also found that different CBCT imaging protocols may introduce different levels of artifacts (Fig. 2) that reduced the accuracy of DIR. Gardner et al. developed a novel iterative reconstruction algorithm to improve CBCT image qualitiy and showed improvements in image uniformity, noise levels, and overall image quality for IGRT for prostate and head and neck cancer.^25^ Tests of the accuracy of DIR for CBCT indicate ongoing room for improvement.

Kujtim et al. pointed out although the AAPM TG 132 report outlines the general goals and criteria for the tests, their specific implementation may be obscure to the wider clinical audience. Moreover, some tests are not accompanied by readily available software to implement them. The literature on validation of image registration software, particularly DIR, is still in its infancy.^26^ Our physical phantom does not have inserts that can simulate all clinical scenerios, but it does provide a means for basic, end‐to‐end testing of the entire imaging system. As noted by Saenz et al., who studied how detailed DIR phantoms need to be to adequately simulate human anatomy and accurately assess the quality of DIR algorithms, concluded that a minimum detail of three levels is a reasonably realistic proxy for use with the Velocity and MIM deformation algorithms.^27^ Our physicial phantom included large and small inserts, which facilitates the need to distinguish soft tissue and bone.

The use of CBCT for proton therapy is evolving as new proton centers are built worldwide.^20^ Verification and adaptive planning should be used during the course of proton therapy for patients with head and neck cancer to ensure that adequate dose is delivered to the planned CTVs while still respecting limits on doses to OARs.^2^ Users of CBCT should ensure that their CBCT system has adequate image quality for daily image guidance of intensity‐modulated proton therapy (IMPT). Recently, Botas et al. developed an online plan adaptation algorithm for IMPT based on fast Monte Carlo dose calculation and CBCT imaging.^28^ Those authors concluded that clinical implementation of their developed algorithm would allow adaptation of dose delivery immediately before treatment, thereby allowing planning margins for IMPT to be reduced. Notably, the correctness of the structures on the CBCTs must be verified; without contour validation, implementing any type of online adaptive therapy would be impossible.

Although significant progress has been made in the past few years in the development of various adaptive radiation therapy techniques, challenges still exist for implementing these techniques in clinical settings.^29^ The accuracy of DIR methods affect the estimates of dose accumulation for both the target dose and the organ dose; one group found consistent correlations between the accuracy of the regions of interest deformation and discrepancies in dose accumulation.^30^ Another group addressed how to accommodate dosimetric variations resulting from anatomic changes during the course of treatments using daily CBCT.^31^ They found that a dose difference of 5% or anatomic variation at a Pearson correlation coefficient of 0.75 indicate practical “action levels” for replanning for the given data sets. In summary, the accuracy of DIR for CBCT in different scanning techniques should be tested before clincial use. Invalid use of the registration results can be a source of error for downstream processes such as contour propagation, dose accumulation, or image guidance.

This study had several limitations. The data presented here should be interpreted as one measure, with a single algorithm, of the effects of CBCT image quality on the accuracy of DIR. We did not attempt to compare different image registration algorithms or different vendor software. All of our test results were based on using Varian Velocity version 4.0 deformable registration software. Other commercial software may well produce different test results depending on the individual DIR algorithm used by each manufacturer. The CBCT acquisition protocols used a sample of available techniques that represent only a few clinical scenarios. A full‐scale evaluation of DIR accuracy with various CBCT image noise and dose levels should be undertaken in the future to investigate additional scenarios.

Finally, use of deformable phantoms for multimodality image registration adds complexity, as it requires phantoms to have components that are optimized for MRI, PET, and single positron emission tomography.^6^ The Wuphantom was designed to faciliate tests of multimodality image registration. The DIR accuracy for other image modalities, either same‐modality or across‐modality (e.g., 4D CT vs CT, MRI vs MRI, MRI vs CT, PET vs PET) have not yet been validated. We plan to continue our studies in these areas in the near future.

## 5 CONCLUSIONS

We quantitatively evaluated the accuracy of DIR for CBCT imaging. The image quality of CBCT images at specified reference CNRs and dose levels can be correlated with the accuracy of the DIR. The Wuphantom facilitates the essential AAPM recommendation that physical phantoms be used for end‐to‐end testing of DIR systems.

## CONFLICT OF INTEREST

Authors Wu, Yang, Wisdom, Liu, Zhu and Frank are inventors on a related pending patent. Other authors have nothing to disclose.
